# A role for TMEM63 in the lung

**DOI:** 10.1172/JCI178948

**Published:** 2024-03-01

**Authors:** Jaime L. Hook

**Affiliations:** 1 Lung Imaging Laboratory, Division of Pulmonary, Critical Care, and Sleep Medicine, Department of Medicine and; 2 Global Health and Emerging Pathogens Institute, Department of Microbiology, Icahn School of Medicine at Mount Sinai, New York, New York, USA.

## Abstract

Surfactants are essential for breathing. Although major progress has been made in the past half century toward an understanding of surfactant secretion mechanisms, the identity of the mechanosensor that couples breathing to surfactant secretion has remained elusive. In this issue of the *JCI,* Chen, Li, and colleagues provide evidence that the mechanosensor is the transmembrane 63 (TMEM63) ion channel. These findings open new avenues for future research into lung mechanobiology.

## Lung surfactants are essential for breathing

Lung surfactants have made respiration possible since our first air-breathing ancestor heaved itself onto a Devonian riverbank. In fact, surfactants are so central to lung function that they are conserved across air-breathing animals, from primitive air-breathing fish to pulmonate snails, reptiles, amphibians, birds, and mammals (1). The centrality of surfactant to lung function lies in its ability to reduce the surface tension of the aqueous liquid layer that lines the lung’s gas exchange surface. Reduction of surface tension weakens the cohesive force that promotes liquid droplet formation, thereby promoting relaxation of the liquid layer against the lung surface and permitting airspaces to be air filled. In humans, the importance of the surfactant system for lung function is underscored by the myriad and profound lung diseases that result from surfactant loss, including pulmonary fibrosis, lung infection, and respiratory distress syndromes across the lifespan.

Although it has been long suspected that a mechanosensor couples surfactant secretion to breathing (2), its identity and character have remained elusive. Surfactant is made in alveolar type 2 (AT2) cells of alveoli, the lung’s gas exchange units. AT2 cells are epithelial cells that, together with AT1 cells, form the alveolar surface. Surfactant is stored in lamellar bodies that orchestrate secretion in response to cellular stimuli. Under baseline conditions, the chief stimulus is breathing. Findings from many groups have generated a model in which inflation-induced distention of the alveolar epithelium causes an increase of cytosolic Ca^2+^ in AT2 cells, leading to cytoskeletal changes that facilitate lamellar body fusion with the plasma membrane and surfactant secretion into airspaces (3–8). Taking these findings together, it becomes clear that the secretion mechanism hinges on the cytosolic Ca^2+^ increase. How does the Ca^2+^ signal arise from the stretch stimulus? What is the mechanosensor that transforms the stretch sensation into a Ca^2+^ response?

## TMEM63: proposed link between breathing and surfactant secretion

In this issue of the *JCI*, Chen, Li, and co-authors suggest that the recently identified transmembrane 63 (TMEM63) ion channel couples lung inflation to increased AT2 cell cytosolic Ca^2+^ and surfactant secretion (9). The proposed mechanism is complex, since the findings do not support the simplest scenario — that is, that TMEM63 would function as a plasma membrane Ca^2+^ channel that opens in response to stretch and facilitates cytosolic Ca^2+^ uptake. Rather, the authors put forth a mechanism in which stretch is sensed by TMEM63 on AT2 cell lamellar bodies, and the TMEM63 response initiates autocrine ATP signaling that leads to cytosolic Ca^2+^ increases and surfactant secretion.

For background, TMEM63 is the animal homolog of the plant “reduced hyperosmolality-induced [Ca^2+^] increase” channel (OSCA) that was first described in 2014 (10). Subsequent work showed that the TMEM63 protein family members TMEM63A, TMEM63B, and TMEM63C function as monomers; TMEM63A and TMEM63B are activated by patch-clamp–mediated negative membrane pressure; and TMEM63B is permeable to numerous cations including Na^+^, K^+^, and Ca^2+^ (11, 12). Recent studies identify a role for TMEM63A and TMEM63B in neurological development and function (11, 13), but the expression and importance of TMEM63 in the lung has not been previously explored.

To investigate the role of TMEM63 in surfactant secretion, Chen, Li, and colleagues carried out studies in mice, isolated lungs, lung slices, and cultured cells (9). The authors found that lung inflation caused an increase of cytosolic Ca^2+^ in AT2 cells that was blocked by AT2 cell–specific codeletion of TMEM63A and TMEM63B. Application of negative pressure to plasma membrane patches of cultured AT2 cells and vacuolin-1–treated lamellar bodies caused cation current activation that was abolished by TMEM63A/B codeletion, suggesting that TMEM63 opened in response to membrane strain. Stretch of cultured AT2 cells caused cellular uptake of the dye FM4-64 in WT cells, but not in cells with TMEM63A/B codeletion. Since uptake of membrane-impermeant, lipophilic pyridinium dyes like FM4-64 marks fusion pores at exocytosis sites, the FM4-64 data indicate that TMEM63A/B was necessary for stretch-induced lamellar body exocytosis. In adult mice, inducible codeletion of TMEM63A and TMEM63B in AT2 cells caused loss of surfactant from bronchoalveolar lavage (BAL) fluid. Taking the findings together, the authors interpret that TMEM63 mediates the inflation-induced cytosolic Ca^2+^ increase in AT2 cells that leads to surfactant secretion.

## Future directions

**Figure 1:**
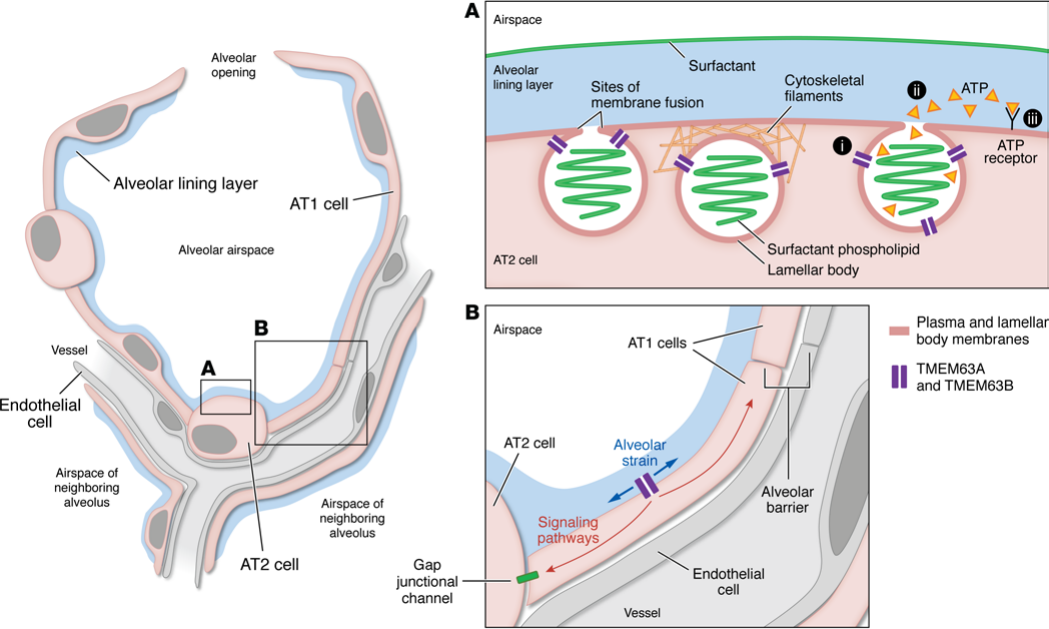
Location and function of TMEM63 in the alveolar epithelium, as proposed by Chen, Li, and co-authors. (**A**) In lung alveoli at homeostasis, lamellar bodies may be found fused with AT2 cell plasma membranes or unfused, and as single vesicles or in chains or clusters. TMEM63 ion channels located on lamellar body membranes might sense AT2 cell stretch through a mechanism termed “force-from-lipids,” in which AT2 cell plasma membrane strain is conveyed to TMEM63 via sites of plasma and lamellar body membrane fusion. Alternatively, or in addition, lamellar bodies may experience what is known as “force-from-filament” mechanotransduction, in which strain is conveyed via plasma membrane–attached cytoskeletal filaments. Chen, Li, and co-authors (9) propose a three-part mechanism by which TMEM63 links inflation-induced alveolar strain to surfactant secretion: (i) stretch-induced activation of cation currents by TMEM63A and TMEM63B causes (ii) lamellar body ATP release and (iii) autocrine ATP signaling, leading to a cytosolic Ca^2+^ increase and surfactant secretion. (**B**) The findings by Chen, Li, and co-authors (9) also suggest that inflation-induced alveolar strain activates TMEM63-mediated cation currents in AT1 cells. Such currents might initiate signaling pathways that regulate alveolar barrier function or promote surfactant secretion in neighboring AT2 cells through, for example, gap junctional communication.

The findings by Chen, Li, and colleagues raise new questions (Figure 1). For example, the authors identified that TMEM63A and TMEM63B localized to the limiting membranes of AT2 cell lamellar bodies, and they patch-clamped AT2 cell lamellar bodies for the first time to show that negative pressure caused cation current activation (9). The lamellar body localization of TMEM63 raises the interesting issue of how lamellar bodies sense cell stretch. Do lamellar body membranes fuse with plasma membranes in response to tidal breathing or sighs, making TMEM63 subject to what is known as “force-from-lipids” (14) mechanotransduction? Or is stretch conveyed to unfused lamellar bodies by cytoskeletal filaments, in a mechanism termed “force-from-filament” (14)? Does lamellar body TMEM63 interact with other ion channels, such as TRPV2 (7)? In applying the patch-clamp findings by Chen, Li, and co-authors to understand alveolar function in vivo, it is important to note that the vacuum stress placed on AT2 cell and lamellar body membranes (9) exceeded the membrane stress expected during physiological inflation of alveoli (15). Going forward, it will be important to ensure that TMEM63 retains its mechanosensitivity under conditions of lesser, more physiological strain. In addition, the spatial distribution of mechanical strain in alveoli is nonuniform (15), and the extent to which AT2 cells or AT2 cell subpopulations stretch with inflation in vivo is not well defined.

Another question relates to the release of lamellar body contents. Chen, Li, and colleagues implicate extracellular ATP, a known stimulator of cytosolic Ca^2+^ increases, in the mechanistic events that connect TMEM63A/B channel activation to surfactant secretion (9). Their findings show that AT2 cell TMEM63A/B deletion decreased extracellular ATP in lung airspaces and that elimination of extracellular ATP in cultured AT2 cells blocked cell stretch–induced Ca^2+^ responses (9). Since ATP is stored in lamellar bodies (16) and released upon cell stretch (17), the authors propose that stretch-induced TMEM63A/B channel activity caused lamellar bodies to release ATP into airspaces, leading to autocrine ATP signaling, cytosolic Ca^2+^ increase, and surfactant secretion (9). It is not clear how lamellar bodies might release ATP without also releasing surfactant at the same time. One possibility is that the rate of ATP release simply outpaces that of surfactant secretion, since studies in isolated lungs show that inflation causes faster release of aqueous components than phospholipid components (8). It would be interesting to better define how the separation between ATP and phospholipid secretion happens. Chen, Li, and co-authors also show that patch-activated currents in HeLa cells transfected with TMEM63B had higher permeability for Na^+^ and K^+^ than Ca^2+^ (9), and they speculate that stretch-induced Na^+^ flux from lamellar body lumens into the AT2 cell cytosol mediated ATP release from lamellar bodies by inducing a lamellar body-shape change. However, direct evidence for this mechanism is lacking, and better understanding is needed of the extent to which lamellar bodies change shape in vivo.

Finally, the findings by Chen, Li, and co-authors raise the interesting question of the function of TMEM63 in AT1 cells. Chen, Li, and colleagues show that, like AT2 cells, AT1 cells express TMEM63 and responded to lung inflation and patch-mediated application of negative membrane pressure with cytosolic Ca^2+^ increases and activation of TMEM63A/B-dependent cation currents (9). These findings align with the view that, although AT1 cells do not contain surfactant, they sense inflation-induced cell strain and initiate cell-cell communication that contributes to surfactant secretion (5, 18, 19). The findings by Chen, Li, and colleagues also suggest that TMEM63 could have a role in function of the alveolar air-blood barrier, which on its epithelial side, is composed primarily of AT1 cells on account of their extensive surface area (20). Thus, the mouse studies by Chen, Li, and colleagues show that inducible TMEM63A/B codeletion in AT2 cells caused pulmonary edema and death (9) and that adding TMEM63A/B deletion in AT1 cells to the AT2 cell TMEM63A/B deletion model accelerated the respiratory failure (9). Better understanding is required of whether TMEM63 regulates the alveolar air-blood barrier.

In sum, the findings reported by Chen, Li, and colleagues provide evidence that TMEM63 is important in lung mechanobiology (9). We need to learn more about TMEM63 expression and function in the alveolar epithelium, particularly whether TMEM63 dysfunction underlies diseases of surfactant deficiency and the extent to which its function might be therapeutically restored.

## Version 1 03/01/2024

Electronic publication
